# DFT + *U* Simulation of the X-ray Absorption Near-Edge Structure of Bulk UO_2_ and PuO_2_

**DOI:** 10.1021/acs.jpcc.3c03143

**Published:** 2023-09-05

**Authors:** Jia-Li Chen, Peter Blaha, Nikolas Kaltsoyannis

**Affiliations:** † Department of Chemistry, University of Manchester, Oxford Road, Manchester M13 9PL, United Kingdom; ‡ Institute of Materials Chemistry, TU Vienna, Vienna A-1060, Austria

## Abstract

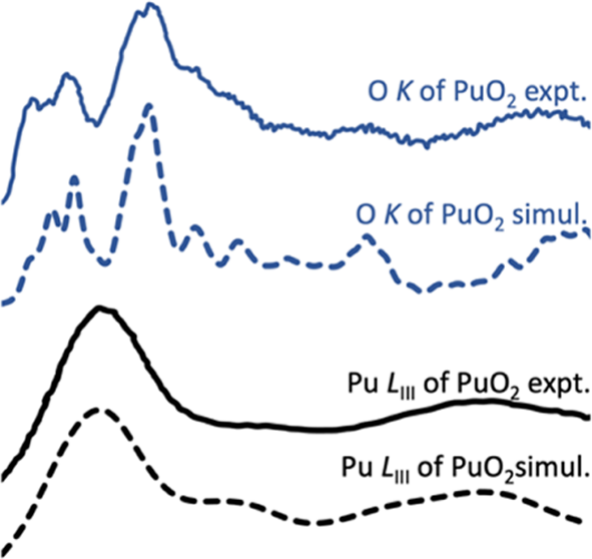

Hubbard *U*-corrected density functional theory within the periodic boundary condition model in the WIEN2k code is used to simulate the actinide *L*_III_ and O *K* edge X-ray absorption near-edge structure (XANES) for UO_2_ and PuO_2_. Spin-orbit coupling effects are included, as are possible excitonic effects using supercells with a core hole on one of the atoms. Our calculations yield spectra in excellent agreement with previous experiments and superior to previous simulations. Density of states analysis reveals the mechanism behind the XANES peaks: the main contribution to the U/Pu *L*_III_ edges comes from the U/Pu *d* states hybridized with O p states, while as expected, the O *p* states primarily determine the O *K* edges of both UO_2_ and PuO_2_. The O *K* edges also feature O *p* hybridizing with U/Pu *d* and *f* states in the low-energy region and with U/Pu *s* and *p* states for the higher-energy peaks.

## Introduction

The actinide dioxides UO_2_ and PuO_2_ are of fundamental interest due in no small part to the fascinating properties of the actinides’ valence 5*f* electrons.^1^ UO_2_ and PuO_2_ are also of major industrial importance because of their central role in the nuclear fuel cycle. The majority of the world’s nuclear reactors use UO_2_ as their fuel, and the recycling of spent Magnox and AGR fuels has, over many decades, separated out significant quantities of Pu, which is currently stored as PuO_2_ powder in the UK. Within this material, the radioactive decay of the Pu has led to the build-up of both U and Am. Good understanding of the evolution of the PuO_2_ on a multidecade timescale is important for its safe storage, and so the study of the fundamental properties of PuO_2_ and Pu/U/Am mixed oxides is essential.

A variety of experimental techniques has been used to explore the electronic structures of UO_2_ and PuO_2_, including X-ray absorption spectroscopy (XAS), electron energy loss spectroscopy (EELS), and photoelectron spectroscopy (PES).^2−7^ Theoretical methods have also been developed to give better predictions of strongly correlated actinide-containing systems,^8^ especially density functional theory (DFT) with Hubbard *U* corrections, which combines reasonable accuracy with computational efficiency, especially in periodic simulations.^9−11^ We are currently part of a joint experiment–theory collaboration, in which one of the principal experimental techniques being employed is X-ray absorption near-edge structure (XANES) spectroscopy, which yields information on both electronic structures and geometries. Theoretical simulation of XANES spectra—the focus of this paper—is important not only to support experimental interpretations but also to provide insight as to the origins of the XANES peaks.

The study of UO_2_ with XANES spectroscopy began decades ago, focusing mainly on the U *L* and *M* edges.^12−14^ These data have recently been enhanced by high-energy-resolution fluorescence detection for X-ray absorption spectroscopy (HERFD-XAS) at the U *L*_III_ edge.^15,16^ The O *K* edge of UO_2_ has also attracted significant interest.^17^ The Pu *L*_III_ and O *K* edges of PuO_2_ are spectroscopically similar to their UO_2_ counterparts.^18−20^ A number of simulations of UO_2_ and PuO_2_ XANES has also been reported.^21−25^ Multiple-scattering calculations within a cluster model reproduce all of the peaks in U *L*_III_ XANES but do not give good predictions of the peak positions and intensities.^21^ The same method does not fare well at the O *K* edge, missing key peaks and giving incorrect positions and intensities for others.^25^ The Pu *L*_III_ edge of PuO_2_ was calculated in the framework of the Anderson impurity model;^26^ the simulation reproduced all of the experimentally reported peaks but did not give good peak positions. It is likely that the failings of the abovementioned simulations result from their being performed on finite clusters, with severe restrictions due to the form of the potential in which the electronic states are calculated. The O *K* edge of PuO_2_ has been studied using a periodic model, but it did not consider core-hole (CH) effects and did not reproduce all of the experimental peaks.^27^

In this work, we present the first systematic simulation of the actinide *L*_III_ and oxygen *K* edges of UO_2_ and PuO_2_ using Hubbard *U*-corrected density functional theory within the periodic boundary condition framework. We show that this approach yields excellent agreement with experiment, significantly superior to previously reported simulations. Furthermore, analysis of the electronic structure of the dioxides allows us to provide detailed interpretations of the origins of the peaks in the edge spectra. Our paper is structured as follows. We first provide computational background and details, before setting out our results for the actinide *L*_III_ edges. We then discuss the oxygen *K* edges, including extended analysis of the effects of the choice of Hubbard *U*, before summarizing our findings in the Conclusions section.

## Computational Background and Details

All XANES simulations have been performed using the periodic WIEN2k code,^28^ and a short introduction to WIEN2k is given in the Supporting Information (SI). The experimental lattice parameters for face centered cubic fluorite UO_2_ and PuO_2_ are used, 5.47 and 5.40 Å, respectively,^29−31^ and the corresponding tetragonal unit cell for the antiferromagnetic (AFM) structure is given in Figure S1a. The 2 × 2 × 1 supercell used for the CH calculations is shown in Figure S1b. A supercell approach is needed since only very few atoms in a solid are actually excited; most remain in their ground state but may contribute to screen the excitonic effects due to the excited core electron on one of the atoms. The generalized gradient approximation of Perdew, Burke, and Ernzerhof (PBE) was chosen,^32^ and a Hubbard *U* correction for the U/Pu 5*f* electrons with a self-interaction correction for the double counting is adopted.^33,34^*R*_MT_ was set to 2.35 au for both U and Pu and 1.96 au for O. The cutoff parameter *R*_MT_*K*_max_ for the basis set is set as 8.5, where *K*_max_ is the cutoff for the basis function wave vector. A Brillouin zone sampling with a 9 × 9 × 6 and 4 × 4 × 7 mesh in the full Brillouin zone is used for the unit cell and supercell, respectively. After testing a wide range of *U* (0.2–0.6 Ry) and *J* (*U*/7–*U*/4) values for ground-state properties such as magnetic moment and the band gap (Figures S2 and S3), a *U* value of 0.3 Ry (4.1 eV) and a *J* value of 0.04 Ry (0.5 eV) were chosen for both UO_2_ and PuO_2_, with the inclusion of spin-orbit coupling (SOC), as these parameters give the best prediction for UO_2_ and PuO_2_ ground-state properties. They also lie in the range of values used in our previous simulations of these materials using the VASP code.^9^ The same value of the Hubbard *U* is added to the An for both An *L* edge and O *K* edge XANES simulations (see below).

Both experiment and previous simulation have found a noncollinear antiferromagnetic (AFM) ground state for UO_2_.^9,35^ By contrast, a nonmagnetic (NM) ground state was found for PuO_2_ from a previous experiment^36^ and our previous simulation, albeit the NM state was obtainable in the latter only by using the occupation matrix control (OMC) approach.^9^ We here use the DFT + *U* method and model both UO_2_ and PuO_2_ with collinear AFM ground states. Comparing the current density of states (DOS, a broadening factor of 0.003 mRy is used for all DOS presented in this work) of AFM PuO_2_ with previous NM simulation, we find that AFM PuO_2_ yields almost the same DOS for the unoccupied states (Figure S3) and, as we shall show, our PuO_2_ XANES simulations also suggest that AFM PuO_2_ is a good approximation for our present purposes. The spin direction of the U/Pu electrons alters between layers to yield the AFM arrangement.

An all-electron method like that implemented in WIEN2k is ideally suited to simulate X-ray absorption spectroscopy. The intensity of the spectrum is given by Fermi’s golden rule according to dipole transitions between initial and final states.^37−39^ For XANES, the final state, which has a CH and an excited electron, determines the spectrum. The CH and the excited electron interact with each other and may lead to strong excitonic effects. The excited electron can be treated as either an additional valence electron (added into the lowest conduction bands) or as a constant background charge to keep the whole system neutral. In some systems, exciting only half a core electron may give better simulations because of incomplete screening effects, and in others, one can obtain good XANES without CHs because of strong screening effects. Although not completely *ab initio* anymore, those parameters should be tested to find the most suitable settings. Therefore, we calculated XANES spectra using a ground-state simulation and a variety of CH calculations: full CH (1CH) and half CH (0.5 CH) are created by removing 1 or 0.5 electrons from U/Pu 2*p* orbitals for the *L*_III_ edge or O 1*s* orbitals for the O *K* edge on one of the atoms in the supercells and adding 1 or 0.5 electrons to the valence band (VB) or background (BG). As our systems are spin-polarized, spin up (up) and spin down (dn) 2*p* or 1*s* electron holes are also compared for 1CH simulations.

## Results and Discussion

Various CH approximations with different spins and excited electron locations are considered for U the *L*_III_ edge XANES of UO_2_, and the resulting spectra are compared in Figure S4a; the spectra are plotted with very low broadening factors *S* = 0.2 eV (spectrometer broadening) and *G* = 1.0 eV (CH lifetime) to yield more detailed XANES. The spectra with different CHs but also those without CH are very similar (Figure S4a). We therefore conclude that the screening of the U 2*p* CH due to the additional occupied 5*f* electron is very strong in bulk UO_2_.

**Figure 1:**
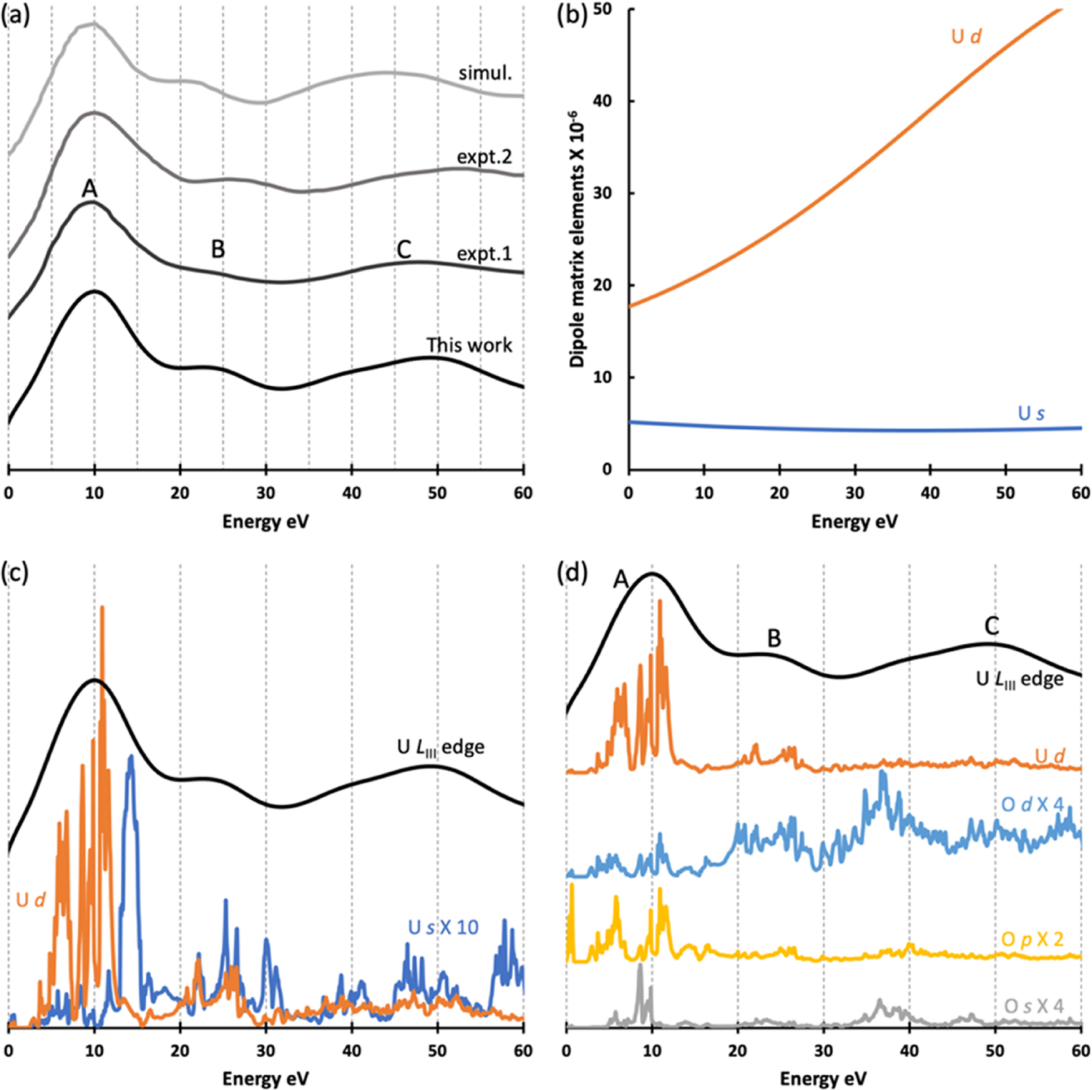
(a) Simulated U *L*_III_ edge XANES of UO_2_ (*S* = 2.4 eV, *G* = 9.0 eV) and previous experimental and simulated spectra: expt.1, expt.2, and simu1 are from refs (14, 16) and (21), respectively; (b) dipole matrix elements of U *s* and *d* states; (c) density of states of U *s* and *d* states and U *L*_III_ edge XANES of UO_2_ (*S* = 2.4 eV and *G* = 9.0 eV); (d) density of states of U d states and amplified O s (4-fold), p (2-fold), and d (4-fold) states, and U *L*_III_ edge XANES of UO_2_ (*S* = 2.4 eV and *G* = 9.0 eV). All calculations are performed without CH. XANES peak A in panels (a) and (d) are aligned and shifted to 10 eV, and the DOS in panels (c) and (d) and dipole matrix elements in panel (b) are also shifted to match the XANES. Spectra and DOS in panels (a), (c), and (d) are shifted vertically for better visibility.

Next, we compare our simulated U *L*_III_ edge of ground-state UO_2_ with two experimental spectra, one XANES and the other HERFD-XAS,^14,16^ and with one simulated spectrum using a cluster approach (the only previous simulation we found).^21^ There are three clear peaks for U *L*_III_ edge XANES, marked as A, B, and C in Figure 1a. expt.2 is the HERFD-XAS spectrum and features clearer peaks with smaller peak widths than expt.1 (XANES) and slightly higher energies for peaks B and C. For comparison with experiment, a broadening factor *S* = 2.4 eV is chosen according to the energy resolution of the previous HERFD-XAS experiment.^16^ A wide range of CH lifetimes *G* (5.0–10.0 eV) are tested, and a value of 9.0 eV gives the best results (Figure S4); it is also close to the 8.67 eV core-hole lifetime of the 2*p*_3/2_ orbital. Our simulation gives almost perfect agreement with experiment for both peak positions and relative intensities (Figure 1a), much better, especially concerning the positions of peaks B and C, than previous cluster modeling results which used the multiple-scattering method.^21^ Overall, for U *L*_III_ edge XANES of UO_2_, we conclude that periodic boundary condition simulation without CH gives accurate results with high computational efficiency.

We further analyzed our simulated spectra to uncover the contributions to the XANES peaks. Motivated by Fermi’s golden rule, we checked the radial transition probability, i.e., the dipole matrix element squared (⟨Ψ_core_|*r*|Ψ_final_⟩^2^), and DOS. Together with the angular factors *W*_1,2_ = 2/5 and *W*_1,0_ = 1, the result gives the energy-dependent transition probability by multiplying the dipole matrix element squared with the corresponding partial DOS. We focus on the unoccupied U *s* and *d* states due to the dipole selection rules. The contribution due to U *s* states is much smaller than U *d* states because the U *s* states have a much smaller dipole matrix (Figure 1b) and much lower DOS (Figure 1c). The DOS of U *d* states are compared with the simulated spectrum in Figure 1d, and the DOS of the O *s*, *p*, and *d* states are also shown. The DOS of the U *d* states are found in the energy range of all three *L*_III_ edge peaks as is hybridization between U d states and O states. Similar DOS shapes are found for U *d* states and O *p* and *d* states around 10 eV. In the energy range 20–30 eV (peak B), clear hybridization between U *d* states and O *p* states can be seen, while from 50 to 55 eV (in the energy range of peak C), we find small double peaks for U *d* states and similar double peaks for O *s* and p states. Overall, it is mainly the U *d* states which contribute to the DOS in the region of the spectral peaks, with hybridization contributions from O states, mainly O *p*.

CH effects at the Pu *L*_III_ edge of PuO_2_ are studied and compared in Figure S5; as with UO_2_, the choice of CH has negligible effect on the spectra, so the screening effects on the Pu 2*p* CH are also very strong. A previous simulation of the Np and Am *L*_III_ edges of NpO_2_ and AmO_2_ also suggested that the CH size does not have much influence on the spectra,^40^ and hence, overall, we conclude that the screening effects to the An 2*p* CH are very strong for AnO_2_ (An = U–Am).

**Figure 2:**
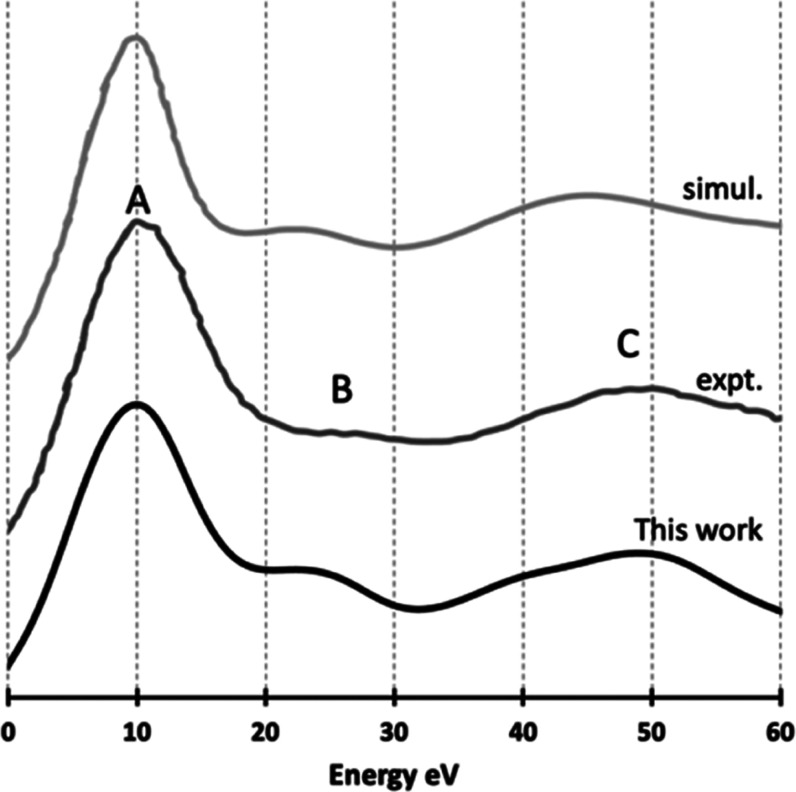
Simulated Pu *L*_III_ edge XANES of PuO_2_ (no CH, *S* = 2.4 eV, *G* = 9.0 eV) and previous experimental and simulated spectra: expt. and simul. are from ref (26). Spectra are shifted vertically for better visibility.

In Figure 2, we compare our ground-state (no CH) simulation with previous HERFD experiment and simulation.^26^ As the energy resolution used is not given in the previous experimental report, the same *S* and *G* values (Figure S5) as used for UO_2_ are employed. As with the U *L*_III_ edge, there are three peaks at the Pu *L*_III_ edge identified by previous experimental XANES, marked as A, B, and C in Figure 2. Our periodic data give almost perfect simulation, for both peak positions and relative intensities, superior to previous cluster modeling results, especially for the positions of peaks B and C; similar improvements were also found for U *L*_III_ above. Overall, our results at the *L*_III_ edge of UO_2_ and PuO_2_ suggest that the best approach to XANES simulation is periodic boundary conditions without CH.

The mechanism underlying the Pu *L*_III_ edge XANES peaks was also studied by analyzing the dipole matrix elements and DOS of the Pu *s* and *d* states (Figure S6); as for the U *L*_III_ edge, the main contribution is from the *d* states. The DOS of the Pu *d* states and those of the O *s*, *p*, and *d* states are compared with Pu *L*_III_ XANES in Figure S6. Pu *d* state DOS are found in the energy range of all three *L*_III_ edge peaks, as well as hybridization contributions from O states, so we conclude that the origin of the *L*_III_ edge XANES peaks for U and Pu in their dioxides is the same.

**Figure 3:**
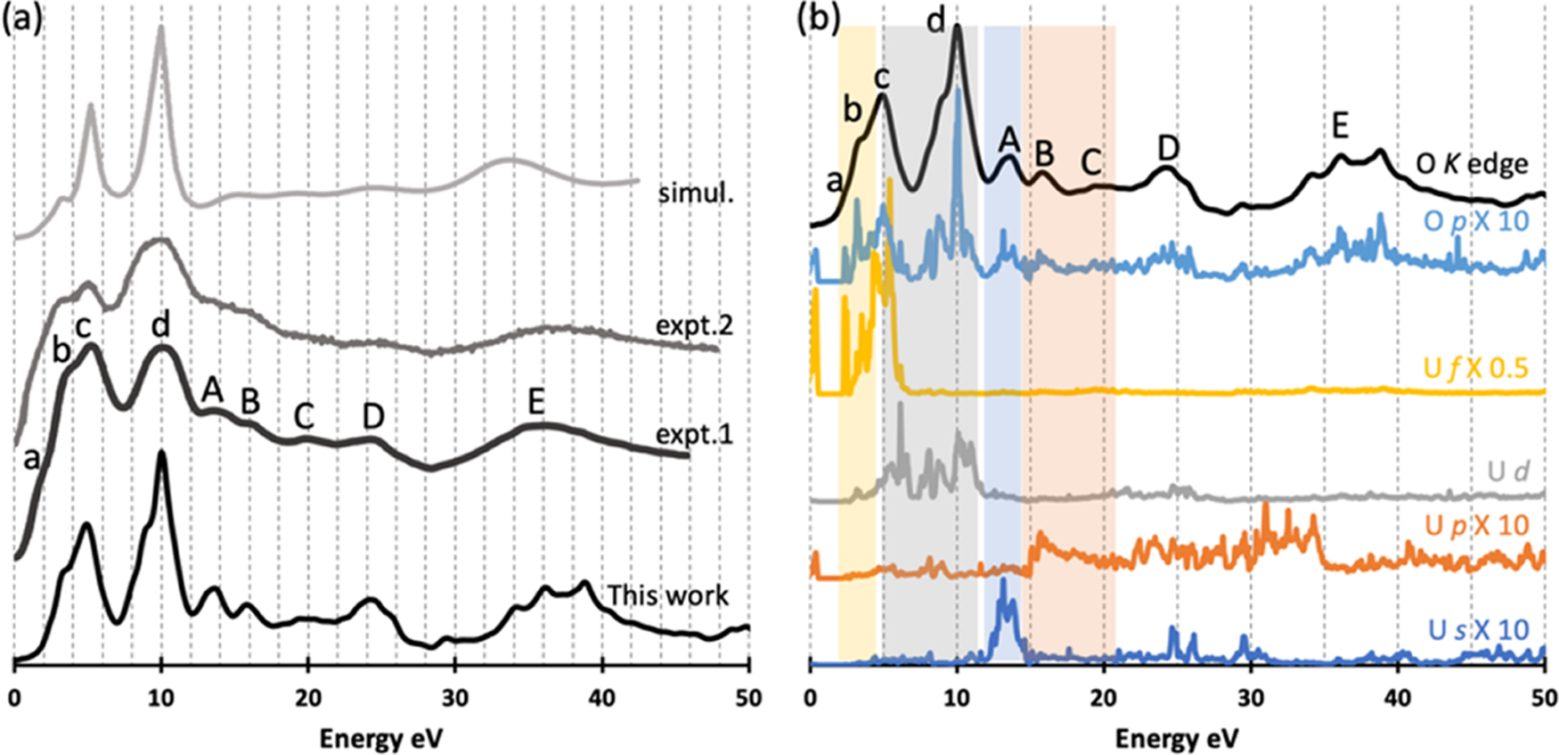
(a) Simulated O *K* edge XANES of UO_2_ for a supercell with 1CH: up-VB (*S* = 0.2 eV, *G* = 0.8 eV) and previous experimental and simulated O *K* edge XANES; expt.1, expt.2, and simul. are from refs (17, 22) and (25), respectively. (b) DOS of amplified O *p* (10-fold) and U *s* (10-fold), *p* (10-fold), and *d* and *f* (0.5-fold) states and O *K* edge of UO_2_ (*S* = 0.2 eV and *G* = 0.8 eV); all simulations are performed with a supercell with 1CH generated by moving one 1*s* electron of O and adding it to the VB. The highest peaks of the XANES are in all figures aligned and shifted to 10 eV. The energy of the DOS is also shifted in panel (b) to match the XANES. Spectra and DOS are shifted vertically for better visibility.

Previous experimental works show that the O *K* edge of UO_2_ features two regions.^17,22^ The first, the up to edge region (0 – 10 eV in Figure 3), has peaks directly connected to the electronic structure, labeled a, b, c, and d. The second, beyond the edge region (>10 eV in Figure 3), features five peaks labeled A, B, C, D, and E—these are related to the crystallographic structure. Similar to the *L*_III_ edges, we first look at the CH effects on the O *K* edge XANES of UO_2_. Ground-state (no CH) and CH simulations of O *K* edge spectra are compared in Figure S7 with *S* = 0.2 eV and *G* = 0.5 eV; we use smaller *G* here than for the *L*_III_ edge, as the lifetime of O 1*s* holes should be larger than that of U 2*p* holes. The CH does not have much influence beyond the edge region, while it does influence the peaks in the lower energy edge region (Figure S7a), so the following discussion focuses on peaks a, b, c, and d. First, we find that the CH has a clear influence on these peaks—the spectrum without a CH has a structure different from the other lines in Figure S7a. Second, the CH size influences the peak intensities and positions (compare 0.5 CH: up-VB with 1CH: up-VB). Finally, the position of the excited electron has a slight influence on the lower energy (<10 eV) peaks; see 1CH: up-VB and 1CH: up-BG in Figure S7a—they are very similar, but the intensities and positions of the first three peaks are slightly different. The initial spin direction (up or down) does not influence the spectra (Figure S7a). Hence, overall, CH effects have clear influence on the O *K* edge XANES of UO_2_, i.e., the screening of the O 1*s* CH is weak in UO_2_ bulk.

**Figure 4:**
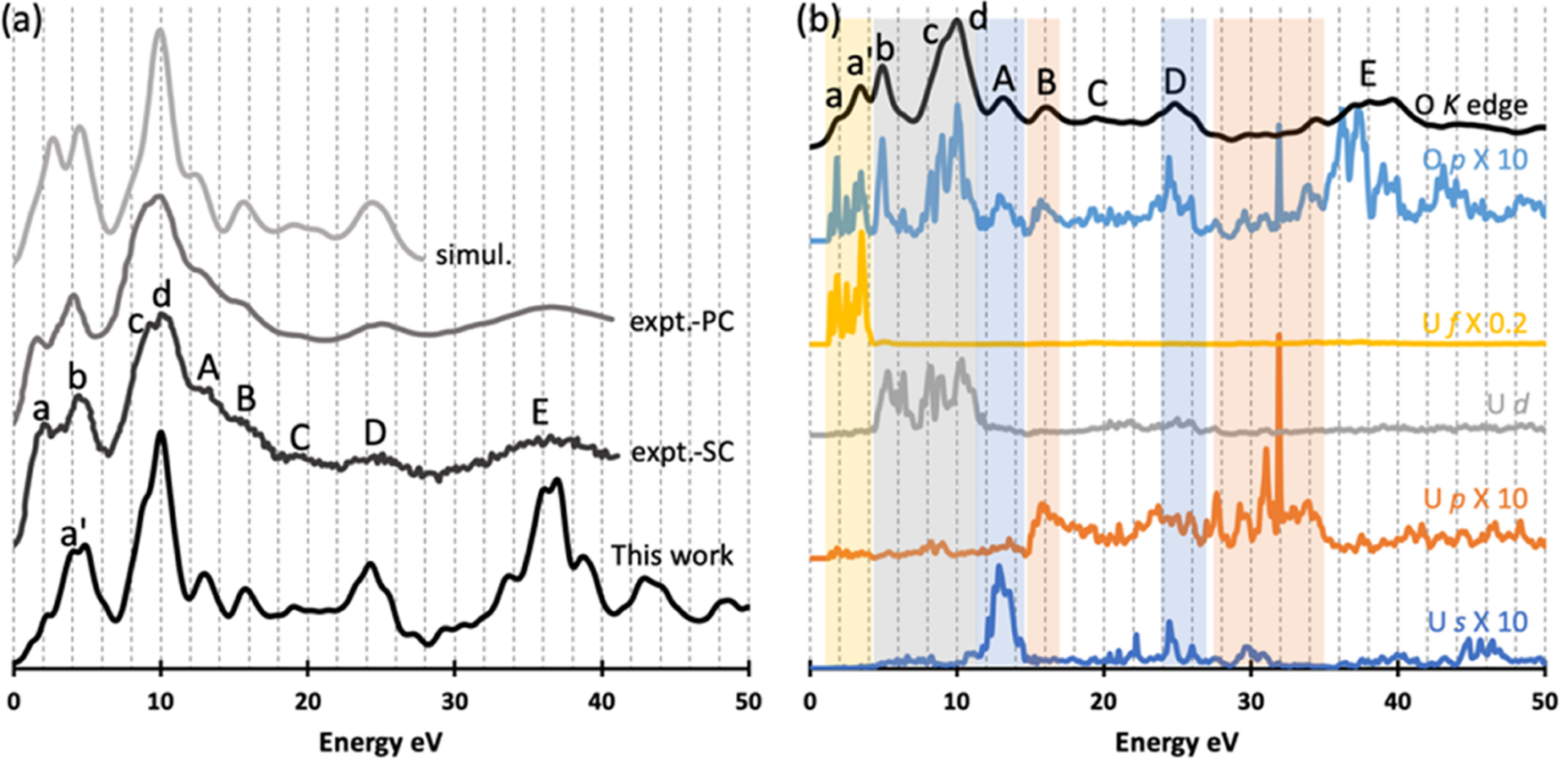
(a) Simulated O *K* edge of PuO_2_ for a supercell with 1CH: up-BG (*S* = 0.5 eV, *G* = 0.6 eV) and previous experimental and simulated O *K* edge data; expt.-SC, expt.-PC, and simul. are from refs (27, 29) to (27), respectively. (b) DOS of amplified O *p* (10-fold) and Pu *s* (10-fold), *p* (10-fold), and *d* and *f* (0.5-fold) states and O *K* edge of PuO_2_ (*S* = 0.5 eV and *G* = 0.6 eV); all simulations are performed with a supercell with 1CH generated by removing one 1*s* electron of O and adding it to the background. The highest peaks of the XANES are in all figures aligned and shifted to 10 eV. The energy of the DOS is also shifted in panel (b) to match the XANES. Spectra and DOS are shifted vertically for better visibility.

We choose 1CH: up-VB simulation to compare with previous experiments and simulation in Figure 3a;^17,22,25^ although 1CH: up-BG also gives a good prediction when compared with experiments, 1CH: up-VB predicts slightly better relative intensities for the first three peaks. The O *K* edge of UO_2_ was studied with energy resolution *S* of 0.2–0.3 eV in previous experiments, so we use *S* = 0.2 eV for our simulated spectrum. The *G* value of 0.8 eV is chosen in the energy range 0.4–5.0 eV (Figure S7b). Our simulation gives a much better spectrum than the previous simulation,^25^ which predicts only three peaks up to the edge region and 4 peaks beyond the edge region and does not do well with the relative intensities of these peaks. Our simulation reproduces all of the experimental peaks (Figure 3a), matching fairly well the peak positions (Table 1) and relative intensities (Figure 3a). The most notable differences are for peak d (a single broad feature in experiment vs. a sharp peak with a shoulder on the left in theory) and for the position of peak a, which is, however, only a weak shoulder in the experiment.

**Table 1:** Simulated Energies of O *K* edge Peaks for UO_2_ (*S* = 0.2 eV, *G* = 0.8 eV) and PuO_2_ (*S* = 0.5 eV, *G* = 0.6 eV), Experimental Data is from References (17, 22) and (27).

	peak (eV)	a/a′	b	c	d	A	B	C	D	E^a^	MAD^b^
UO_2_	expt.1	1.55	3.65	5.2	10.0	13.9	16.1	19.85	24.85	37.05	0.319
	expt.2	1.6	3.4	5.2	10.0	14.0	16.1	19.8	24.5	36.9	0.309
	this work	2.36	3.20	4.96	10.00	13.76	15.88	19.90	24.62	37.78	
PuO_2_	expt.	2	4.4	9.3	10	12.7	15.5	19.7	25.1	37	0.507
	this work	1.78/3.41	4.92	8.96	10.00	13.14	16.06	19.42	24.82	38.92	

a The position of high-energy peak E is obtained from a simulated spectrum with a lifetime broadening factor *G* = 4.0 eV.

b The mean absolute deviation (MAD) of our simulations from the available experimental data are given in the last column.

We further studied the O *K* edge XANES by analyzing the DOS of the unoccupied states. Due to the dipole selection rule, only O *p* states contribute directly to the spectrum, but to emphasize possible hybridizations of O *p* with U *s*, *p*, *d*, and *f* states, their partial DOS are also shown in Figure 3b. In the low-energy region, there is clear hybridization between O *p* and U *f* states up to 6 eV (highlighted with a yellow background in Figure 3b) that contributes to peaks a, b, and c. Hybridization between O *p* and U *d* states (gray background) shows U *d* state contribution to peak c and in particular d. Beyond the edge region, peak A arises from the hybridization between O *p* and U *s* states (blue background), peaks B and C come from the hybridization between O *p* and U *p* states (orange background), while peaks D and E mainly come from O *p* states, perhaps with some hybridization from U *s* and *p* states. Overall, it is the intensities and positions of the O *p* states which determine the O *K* edge peaks, modified by hybridization with U *d* and *f* states in the up to edge region and having contributions from U *s* and *p* states in the region beyond the edge.

Similar to the O *K* edge of UO_2_, previous experiments show that the O *K* edge of PuO_2_ can be divided into two parts: up to the edge region (0–10 eV in Figure 4) with four main peaks a, b, c, and d and beyond the edge region (>10 eV in Figure 4) with five peaks A, B, C, D, and E.^19,27,41^ We again begin by examining the CH effects. Ground-state (no CH) and CH simulations of O *K* edge spectra are compared in Figure S8a; as with UO_2_, CH effects have a clear influence on the O *K* edge XANES of PuO_2_, so the screening effect of the valence electrons on the O 1*s* CH is not very strong.

A full CH generated by removing one O 1*s* electron and adding it to background gives the best simulation results, so we focus on the 1CH: up-BG spectrum. In Figure 4, the two experimental spectra are from single-crystal ^239^PuO_2_ (line expt.-SC) and polycrystalline ^242^PuO_2_ (line expt.-PC), and their energy resolution is not larger than 0.6 eV. For better comparison with the experiments, we use an *S* value of 0.5 eV and a *G* value of 0.6 eV in the range of 0.4–5.0 eV (Figure S8b). We have discovered one previous simulation, also performed in WIEN2k, though using only the ground-state electronic structure and not considering CH effects.^27^

Our simulation reproduces all of the experimental peaks. Beyond the edge region, we obtain very good positions and relative intensities for peaks A, B, C, D, and E compared with the experiment (Table 1 and Figure 4a). A previous calculation without CH^27^ (line simul. in Figure 4a) also yields all of the peaks beyond the edge region with a reasonable position and intensity. Thus, the CH effect has only a slight influence on the beyond edge peaks, as is the case for the O *K* edge of UO_2_ (Figure S7). In the up to the edge region, CH simulation does not predict a sufficient number of peaks (line simul. in Figure 4b), while our simulation reproduces all of the experimental peaks, once again proving the importance of CH on the simulation of the O *K* edge. Our simulation predicts a peak a′ (around 3–4 eV) between peaks a and b, which seems to be also present in the experimental results, especially the single-crystal PuO_2_ experiment but was not discussed in the experimental work.^27^

The DOS of PuO_2_ are studied to gain further insight into the XANES peaks, especially for the up to the edge region peaks. O *p* states (dipole selection rule) and Pu *s*, *p*, *d*, and *f* states (hybridization) are compared with our simulated XANES in Figure 4b. The O p states clearly determine the XANES peaks. In the low-energy region, the Pu *f* states hybridize with O *p* states and contribute to peaks a and a′ (yellow background in Figure 4b), while the Pu *d* states hybridize with O *p* states and contribute to peaks b, c, and d (gray background in Figure 4b). Beyond the edge region, hybridization contributions from Pu *s* and *p* states are highlighted with blue and orange backgrounds, respectively, in Figure 4b.

There are some small differences between our simulations and previous experiments for the O *K* edges. For example, in Figure 3a, experiment finds a round peak d for UO_2_, while our simulation predicts a sharper peak, with a left shoulder peak around 9 eV. Also, the relative intensities and positions of peaks a, b, and c at the UO_2_ O *K* edge are slightly different from experiment (Table 1). In Figure 4a, there is a clear peak a′ at about 3 eV in our simulated O *K* edge of PuO_2_, though this feature is observed only weakly in the experimental results, and our simulation predicts much lower intensity for peak a at the PuO_2_ O *K* edge. We wondered if these small differences could arise from our simulation method, specifically the choice of *U* in the GGA + *U* approach or possibly choosing GGA + *U* over hybrid DFT. However, as we show in Figures S9 and S10, the *U* value has negligible influence on the peaks’ position and intensity, and DFT + *U* simulations predict almost the same spectra as hybrid DFT simulations.

## Conclusions

In this contribution, we report periodic DFT + *U* + SOC simulations of the actinide *L*_III_ and O *K* edges of bulk UO_2_ and PuO_2_. Detailed assessment of computational parameters such as the choice of *U* and *J* and the treatment of core-hole effects allow us to generate simulated spectra that are in excellent agreement with experimental data. Core-hole effects have little influence on the actinide *L*_III_ edges due to the strong screening effect of the valence electrons, and hence using a unit cell without a core hole works well. By contrast, O 1*s* core holes, which are not fully screened by the valence electrons, have clear influence on the O *K* edge XANES, mainly on the low-energy, electronic structure-related peaks, and we recommend the use of a full core hole with a supercell for accurate O *K* edge simulation.

The excellent performance of the methodology employed here within the WIEN2k code gives us much confidence in our simulations, and we are now moving to apply the same approach to study other edges, such as the An *M* and *N*, and more complex actinide oxides, e.g., the actinide *L*_III_ and O *K* edges of Pu/U/Am mixed dioxides.

## Data Availability

The data supporting the findings reported in this paper are openly available at DOI: 10.17632/v2x87s8sy9.1.

## Supporting Information Available

Additional computational details, parameter settings, and simulated spectra (PDF)
