# Supplementary material for: DFT + U Simulation of the X-ray Absorption Near-Edge Structure of Bulk UO2 and PuO2
Source: J Phys Chem C Nanomater Interfaces. 2023 Sep 5;127(36):17994–8000. doi: 10.1021/acs.jpcc.3c03143 (PMC10510436; doi:10.1021/acs.jpcc.3c03143)
Supplement: Supplementary file 1 — jp3c03143_si_001.pdf [file jp3c03143_si_001.pdf]

# DFT + *U* Simulation of the X-ray Absorption Near-edge Structure of Bulk $UO_2$ and $PuO_2$

Jia-Li Chen,[a,*] Peter Blaha[b] and Nikolas Kaltsoyannis[a]

[a] Department of Chemistry, University of Manchester, Oxford Road, Manchester M13 9PL, United Kingdom

[b] Institute of Materials Chemistry, TU Vienna, A-1060 Vienna, Austria

ORCID
Jia-Li Chen: 0000-0003-2964-0127
Nikolas Kaltsoyannis: 0000-0003-0293-5742

* Email: jiali.chen@manchester.ac.uk

## Supporting Information

## The WIEN2k programme

WIEN2k is based on the augmented plane wave plus local orbitals (APW + LO) method[1-2] to solve the Kohn-Sham equations of density functional theory (DFT).[3-4] The unit cell is divided into two regions, non-overlapping atomic spheres that are centred at the nuclear sites, and the interstitial region.[5] The atomic sphere radii ($R_{MT}$) are set automatically, depending on the geometry and the atomic species. The electronic states are decomposed into core and valence states. Core states are defined as having wave functions (densities) which are completely confined inside the atomic spheres; these are constrained to be localized, do not hybridize with neighbouring atoms' states but are recalculated in each self-consistent field (SCF) cycle. The valence electrons have basis functions consisting of APWs, which are plane waves in the interstitial region augmented with an angular momentum expansion with numerical radial wavefunctions $u_\ell(r,E)$ defined at a fixed energy, and LOs which contain the energy derivative of $u_\ell$ to allow the necessary variation of the radial functions. Additional LOs, which contain radial functions expanded at an appropriate energy, are added to treat states having lower energies than the valence states, which are called semi-core states. All energy parameters are chosen automatically and dynamically updated during the SCF cycle. Spin-orbit coupling (SOC) can be included in a second variational step using the scalar relativistic orbitals as a basis.[4, 6] Numerous density functionals are implemented in WIEN2k, such as LDA, GGA, meta-GGA and hybrids,[7] while for strongly correlated electrons DFT+$U$ is also available.[8-9]

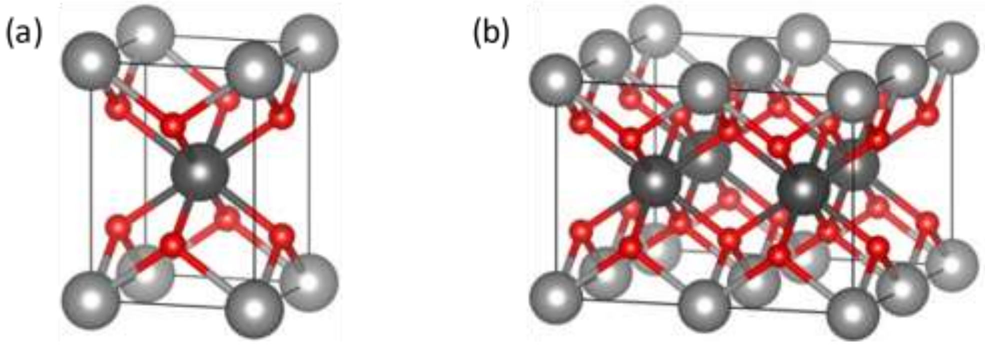


Figure S1: (a) unit cell of AFM $UO_2$ or $PuO_2$ (b) 2 X 2 X 1 supercell of AFM $UO_2$ or $PuO_2$. Grey and red balls are U/Pu and O, respectively, light grey U/Pu denotes dominance of spin up, dark grey U/Pu of spin down electrons.

## Searching for optimum *U* and *J*

A wide range of $U$ values (0.2 – 0.6 Ry) are tested for AFM $UO_2$ unit cell; band gap and magnetic moment of U atoms are compared with experiments to search for a suitable $U$ value. Spin-orbit (SO) coupling has negligible influence on band gap (Figure S2 (a)), while has obvious influence on magnetic moment on U atoms (Figure S2 (b)). Without SO coupling, calculations predict slightly higher magnetic moment for U atoms than the experimental value 1.74 $\mu_B$,[10] and the magnetic moment (including spin moment and orbital moment) of U decreases by about 0.4 $\mu_B$ by taking SO coupling into consideration. We then did the same calculations with $J$ ($U/7 – U/4$) (Figure S2 (c) and (d)), as 0.3 Ry ≤ $U$ ≤ 0.4 Ry predict close band gaps for $UO_2$ to experimental range, $J$ values are only tested for $U$ = 0.3, 0.35 and 0.4 Ry with SO coupling. Influence of $J$ value on band gap is negligible (Figure S2 (c)), while largely increases the magnetic moment on U atoms (Figure S2 (d)), which increases with $J$ values. With $U$ = 0.3 Ry (4.1 eV) and $J = U/7$ = 0.04 Ry (0.5 eV), a band gap of 2.25 eV and magnetic moment of 1.73

$\mu_B$ for U atoms are predicted, very close to the experimental values. The effective *U* value (3.6 eV) is also in the range of our previously suggested *U* values for $UO_2$ bulk simulations.[11]

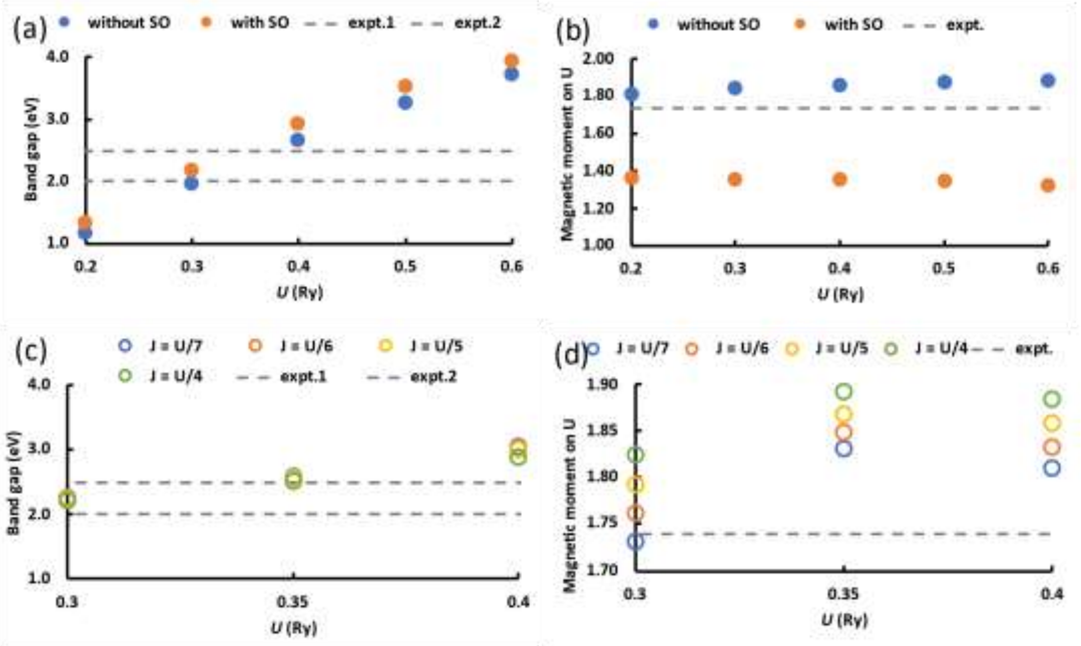


Figure S2: (a) band gap and (b) magnetic moment on U atoms for $UO_2$ calculated with PBE + *U* without and with spin-orbit (SO) coupling; (c) band gap and (d) magnetic moment on U atoms for $UO_2$ calculated with PBE + *U* + *J* with spin-orbit (SO) coupling.

We also adopt an AFM state for $PuO_2$, as it is predicted as the lowest energy state with the DFT + *U* method, although previous experiments and our previous DFT + *U* + OMC simulation support the nonmagnetic ground state for $PuO_2$. As we will consider the core-hole effects, the ground state is different for $PuO_2$ with and without core-hole. Besides, the density of states (DOS) of unoccupied states is the most important thing we need to take into consideration for XANES simulation. Therefore, we use AFM $PuO_2$ in this work and search a *U* value by DOS. The *U* = 0.3 Ry give the best DOS,[11] so *U* = 0.3 Ry (4.1 eV) and *J* = *U*/7 = 0.04 Ry (0.5 eV) is used in this work for all $PuO_2$ simulations.

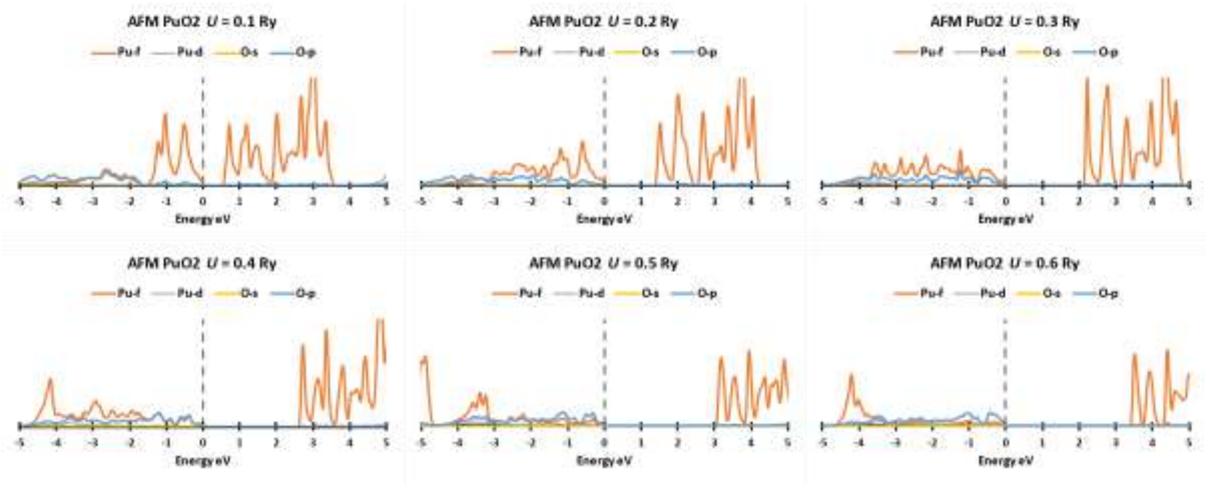

Figure S3: density of state (DOS) of AFM $PuO_2$, simulated with *U* value range from 0.1 Ry to 0.6 Ry and $J = U/7$.

## U/Pu $L_{III}$ edge

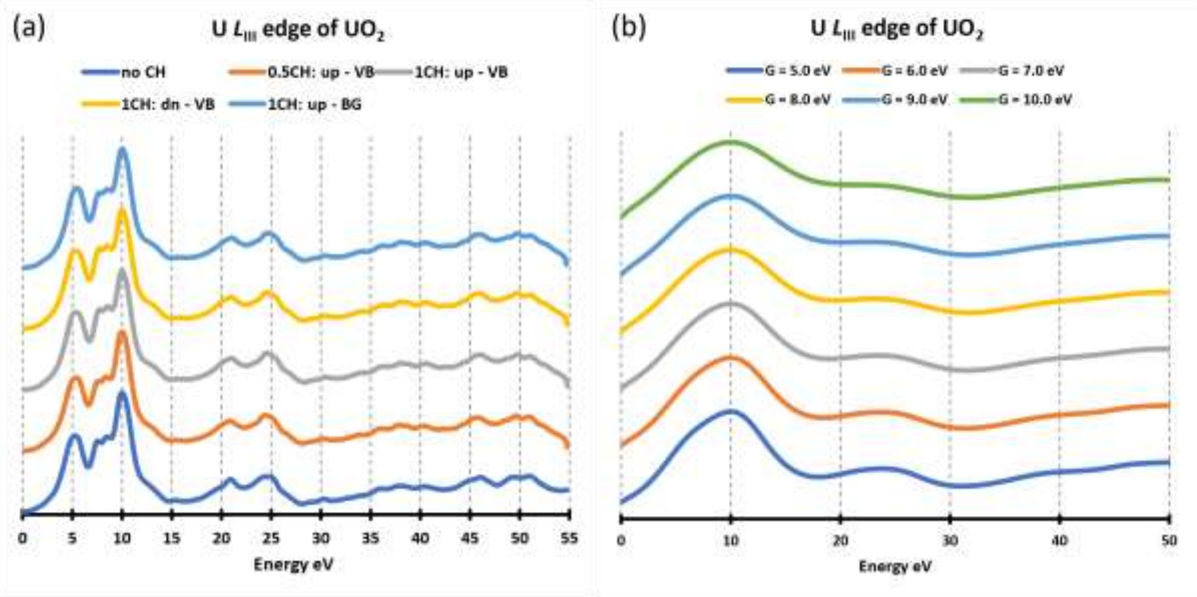


Figure S4: (a) simulated *U* $L_{III}$ edge of ground state $UO_2$ (unit cell) and with different core-hole treatment with a spectrometer broadening factor *S* = 0.2 eV and G= 1.0, (b) ground state calculation with life-time broadening factor *S* = 2.4 eV and *G* as given in the legend. The highest peaks of XANES in the figures are aligned and shifted to 10 eV, spectra are shifted vertically for better visibility.

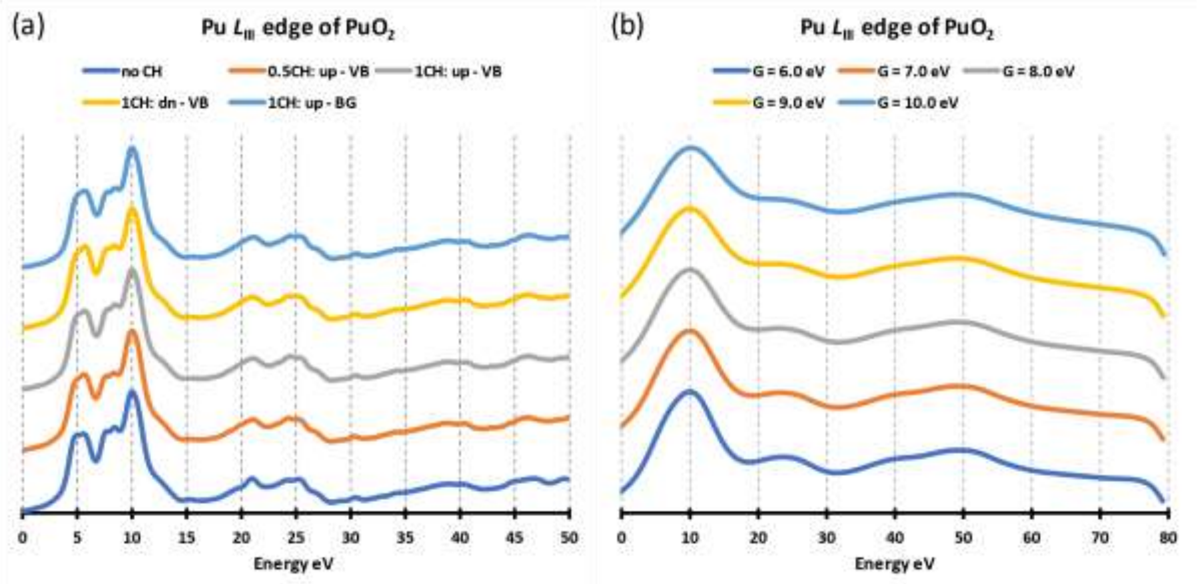


Figure S5: (a) simulated Pu $L_{III}$ edge of ground state $PuO_2$ (unit cell) and with different core-hole treatment with a spectrometer broadening factor *S* = 0.2 eV and *G* = 1.0 eV. (b) ground state calculation with life-time broadening factor *S* = 2.4 eV and *G* as given in the legend. The highest peaks of XANES in figures are aligned and shifted to 10 eV, the spectra are shifted vertically for better visibility.

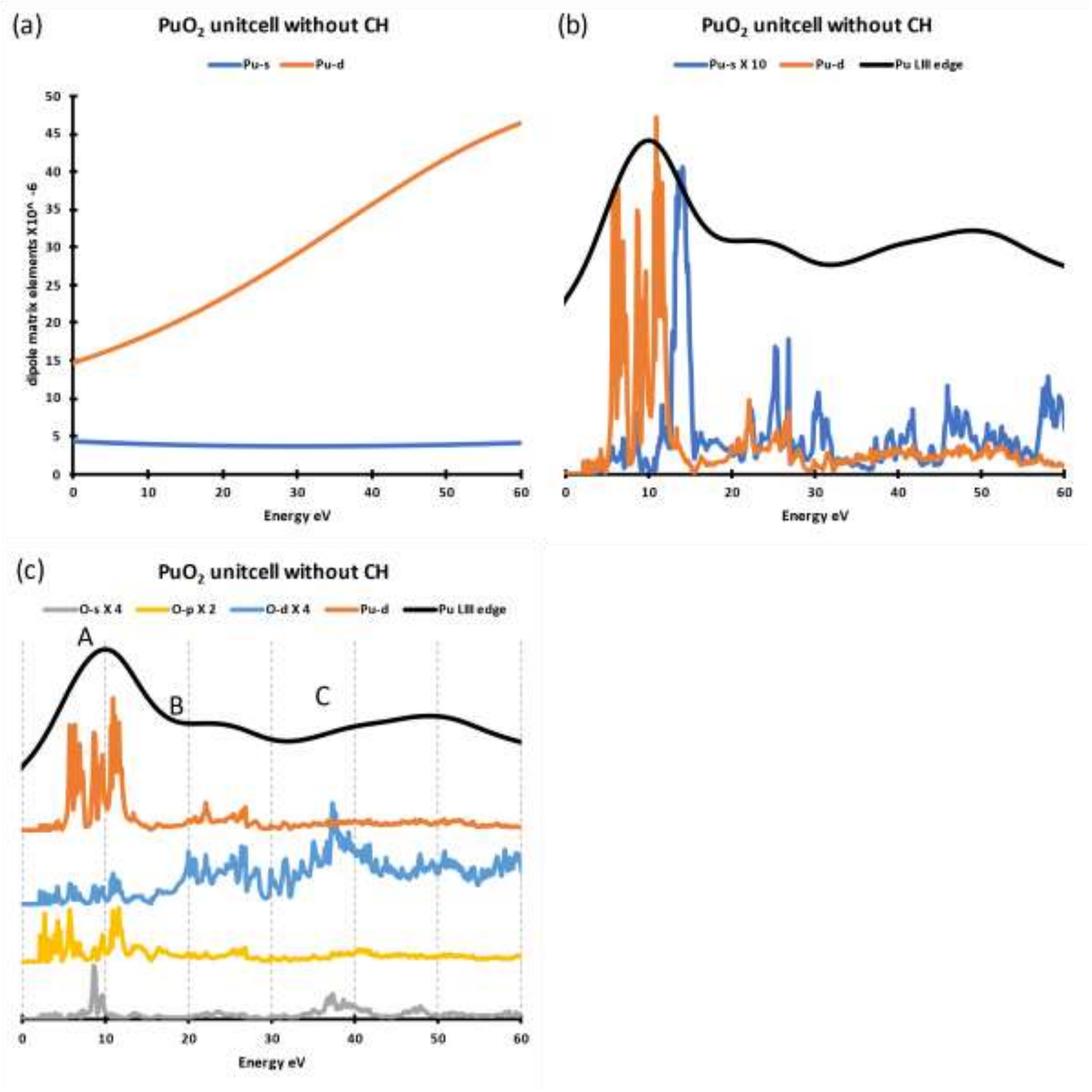


Figure S6: (a) dipole matrix elements of Pu *s* and *d* states; (b) density of state (DOS, broadening factor is 0.003 mRy) of Pu *s* (10 fold amplified) and *d* states and Pu $L_{III}$ edge XANES of $PuO_2$ with *S* = 2.4 eV and *G* = 9.0 eV. (c) DOS of Pu *d* states and amplified O *s* (4-fold), *p* (2-fold) and d (4-fold) states with a broadening factor of 0.003 mRy, and Pu $L_{III}$ edge XANES of $PuO_2$ with *S* = 2.4 eV and *G* = 9.0 eV. All calculations are done without CH. The highest XANES peak in figure (b) and (c) is shifted to 10 eV, the energy of DOS in figure (b) and (c) and dipole matrix elements in figure (a) are also shifted to match the XANES. Spectra and DOS in figure (b) and (c) are shifted vertically for better visibility.

## O *K* edge

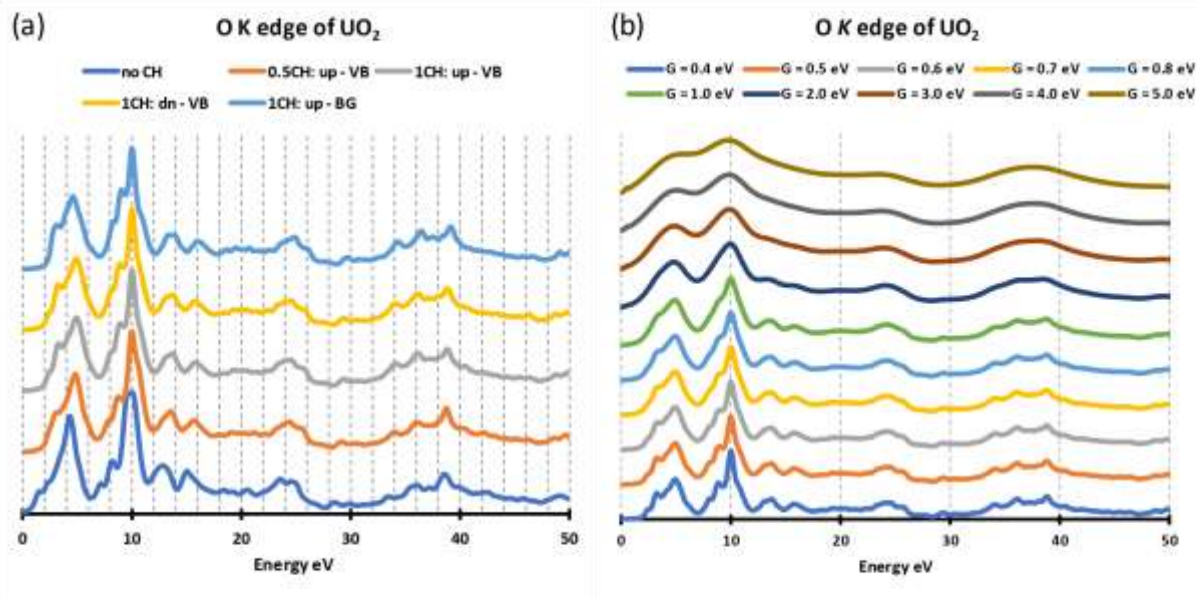


Figure S7: (a) simulated O *K* edge of $UO_2$ with different core-hole treatment and broadening factor *S* = 0.2 eV and *G* = 0.5 eV. (b) simulated O *K* edge of $UO_2$ for supercell with 1 CH: up-VB; broadening factor *S* = 0.2 eV and *G* is given in the legend. The highest peaks of XANES in figures are aligned and shifted to 10 eV, the spectra are shifted vertically for better visibility.

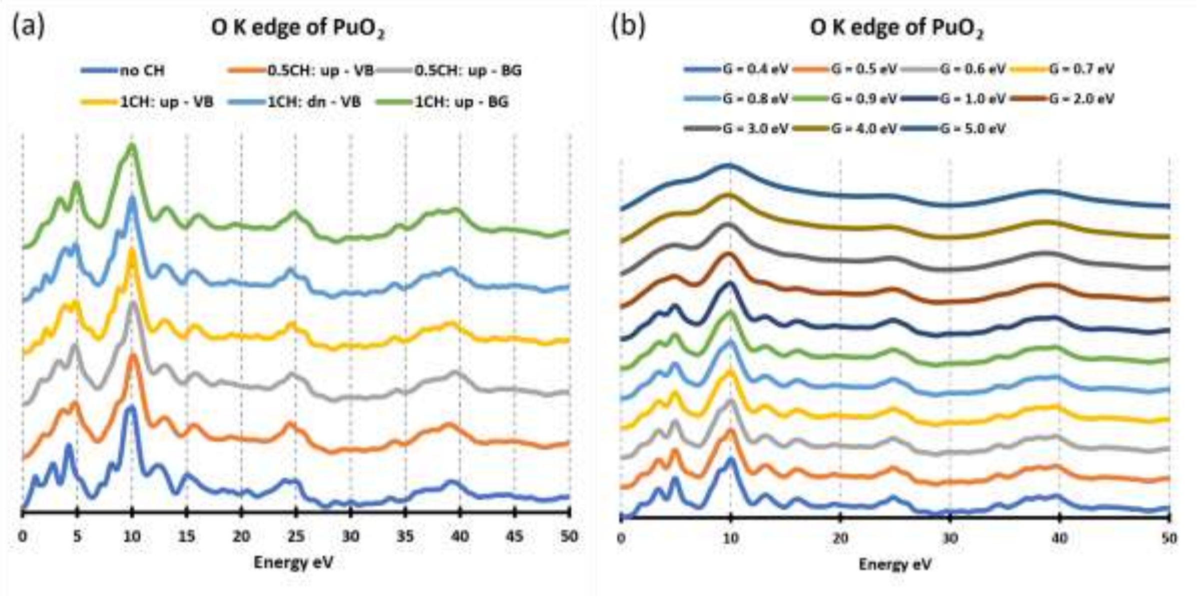


Figure S8: (a) simulated O *K* edge of $PuO_2$ with different core-hole treatment and broadening factor *S* = 0.2 eV and G = 0.5 eV. (b) simulated O *K* edge of $PuO_2$ for supercell with 1 CH: up-BG; with broadening factor *S* = 0.5 eV and G is given in the legend. The highest peaks of XANES in figures are aligned and shifted to 10 eV, the spectra are shifted vertically for better visibility.

The choice of *U* has little influence on the beyond edge peaks in the O *K* edge XANES for both $UO_2$ and $PuO_2$; the peaks' positions (Table S1) and relative intensities (Figure S9) are very close for XANES simulated with different *U* values. In the lower energy region, the overall structures of the simulated spectra also do not show much dependence upon *U*, *e.g.* peak d of the $UO_2$ O *K* edge is always sharp in our simulated spectra, and the left shoulder peak around 9 eV

does not disappear with larger or smaller *U* values. Similarly, peak a' in the $PuO_2$ O *K* edge XANES is found in all simulated spectra (Figure S9 (b)). However, there is some effect of *U* on the positions and relative intensities of the lower energy peaks. The relative intensities of peaks a and b of $UO_2$ and peaks a and peak a' of $PuO_2$ increase with *U* value. All up to the edge peaks move to higher energies with increasing *U* value. *U* = 0.3 Ry, which was chosen based on the ground state properties, also seems most appropriate for the O *K* edge, as simulations with this *U* value give the smallest mean absolute deviations with the experimental peak positions. Overall, we conclude that the choice of *U* slightly influences the intensities and positions of the low energy peaks at the O *K* edge of $UO_2$ and $PuO_2$, but this variation is not sufficient to eliminate the small differences between our simulations and previous experiments.

Table S1: energies of the O *K* edge peaks for $UO_2$ (*S* = 0.2 eV, *G* = 0.8 eV) and $PuO_2$ (*S* = 0.5 eV, *G* = 0.6 eV), Our simulations are performed with different *U* values (*J* = *U*/7) for a supercell with 1CH, generated by moving one spin up 1*s* electron of O to the valence band or to background for $UO_2$ and $PuO_2$, respectively. All XANES are shifted such that all peaks d are at 10.0 eV. The mean absolute deviation (MAD) of our simulations from the experimental data (Table 1) are given in the last column.

| Peak (eV) | | a | b | c | d | A | B | C | D | E* | MAD |
|---|---|---|---|---|---|---|---|---|---|---|---|
| UO2 | 0.40 Ry | 2.53 | 3.41 | 5.09 | 10.00 | 13.59 | 15.81 | 20.43 | 24.21 | 36.79 | 0.379/0.309 |
| | 0.30 Ry | 2.36 | 3.20 | 4.96 | 10.00 | 13.76 | 15.88 | 19.90 | 24.62 | 37.78 | 0.319/0.307 |
| | 0.20 Ry | 2.16 | 2.92 | 4.70 | 10.00 | 13.72 | 15.82 | 19.84 | 24.62 | 37.56 | 0.339/0.324 |
| PuO2 | 0.40 Ry | 2.22 | 5.10 | 9.04 | 10.00 | 13.20 | 16.20 | 19.52 | 24.92 | 39.98 | 0.636 |
| | 0.30 Ry | 1.78 | 4.92 | 8.96 | 10.00 | 13.14 | 16.06 | 19.42 | 24.82 | 38.92 | 0.507 |
| | 0.20 Ry | 1.40 | 4.88 | 8.90 | 10.00 | 13.14 | 16.02 | 19.34 | 24.74 | 38.74 | 0.544 |

* The position of high energy peak E is obtained from a simulated spectrum with a lifetime broadening factor *G* = 4.0 eV

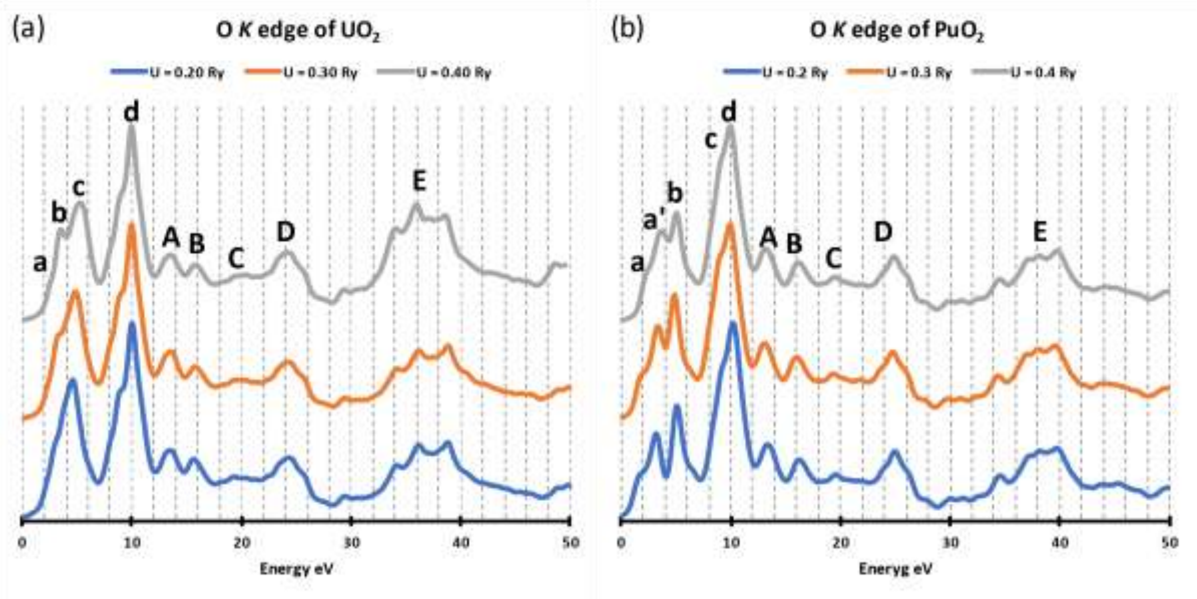


Figure S9: (a) simulated O *K* edge XANES with different *U* value for $UO_2$ supercell with 1 CH: up-VB (*S* = 0.2 eV, *G* = 0.8 eV); (b) simulated O *K* edge XANES with different *U* value for $PuO_2$ supercell with 1 CH: up-BG (*S* = 0.5 eV, *G* = 0.6 eV). The highest peaks of the XANES in all figures are aligned and shifted to 10 eV, intensities are scaled to 1, and spectra are shifted vertically for better visibility.

## Hybrid-DFT simulations for O *K* edge:

We also performed some hybrid DFT simulations for the O *K* edge of $PuO_2$, with and without CH (1CH: up – BG), using the Yukawa-screened hybrid functional YS-PBE0,[12-13] which is very similar to the well-known HSE functional.[14-15] The screening parameter $\omega$ in YS-PBE0 is set to 0.165 $\lambda$ (the screening length in HSE), but uses an exponential instead of the error function for screening in WIEN2k. In hybrid functionals a fraction $\alpha$ of semi-local (SL) exchange is replaced by Hartree-Fock (HF) exchange:

$$E_{xc}^{hybrid} = E_{xc}^{SL} + \alpha(E_x^{HF} - E_x^{SL})$$

We retained PBE for the SL exchange and a value of 0.25 for $\alpha$. We first performed the hybrid simulations with a reduced *k*-mesh (2 X 2 X 3 for the supercell), then redid the calculations from the converged calculation with a finer 4 X 4 X 6 *k*-mesh. Hybrid DFT simulations are compared with DFT + *U* simulations in Figure S10. To save computational time, only the low energy regions were studied, as the differences are mainly found in this region. The hybrid and DFT + *U* simulations predict almost the same structure for the up to edge peaks, with and without CH.

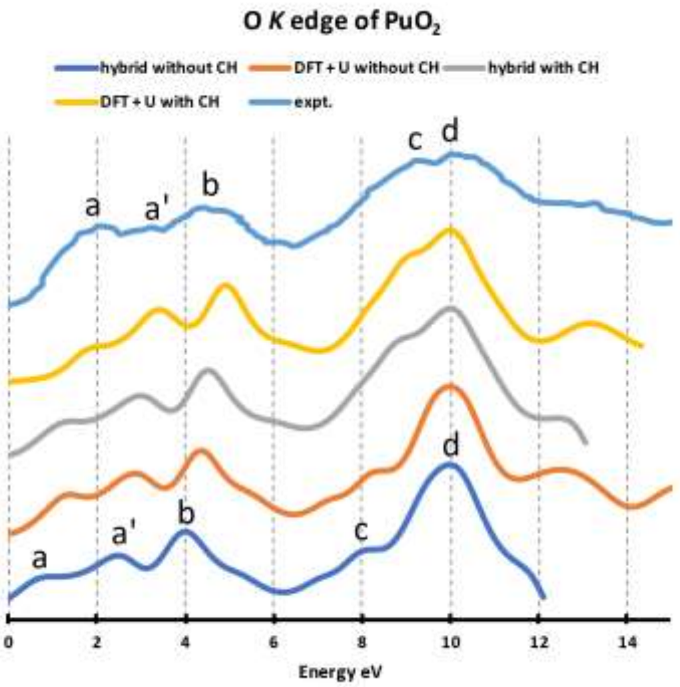


Figure S10: simulated O *K* edge of $PuO_2$ supercell (*S* = 0.5 eV, *G* = 0.6 eV), and previous experimental O *K* edge of $PuO_2$ (reference). For CH simulation, the CH is generated by removing a O 1*s* electron and adding to the background charge.

## References


1. Sjöstedt, E.; Nordström, L.; Singh, D. J., An alternative way of linearizing the augmented plane-wave method. *Solid State Communications* **2000**, *114*, 15-20.
2. Madsen, G. K.; Blaha, P.; Schwarz, K.; Sjöstedt, E.; Nordström, L., Efficient linearization of the augmented plane-wave method. *Physical Review B* **2001**, *64*, 195134.
3. Hohenberg, P.; Kohn, W., Inhomogeneous electron gas. *Physical review* **1964**, *136*, B864.
4. Kohn, W.; Sham, L. J., Self-consistent equations including exchange and correlation effects. *Physical review* **1965**, *140*, A1133.
5. Singh, D. J.; Nordstrom, L., *Planewaves, Pseudopotentials, and the LAPW method*; Springer Science & Business Media, 2006.
6. MacDonald, A.; Picket, W.; Koelling, D., A linearised relativistic augmented-plane-wave method utilising approximate pure spin basis functions. *Journal of Physics C: Solid State Physics* **1980**, *13*, 2675.
7. Perdew, J.; Schmidt, K., Jacob's ladder of density functional approximations for the exchange-correlation energy AIP Conf. *Proc. 577* **2001**, *1*.
8. Anisimov, V. I.; Solovyev, I.; Korotin, M.; Czyżyk, M.; Sawatzky, G., Density-functional theory and NiO photoemission spectra. *Physical Review B* **1993**, *48*, 16929.
9. Laskowski, R.; Madsen, G. K.; Blaha, P.; Schwarz, K., Magnetic structure and electric-field gradients of uranium dioxide: An ab initio study. *Physical Review B* **2004**, *69*, 140408.
10. Faber Jr, J. In *Magnetic moment distribution in uranium dioxide*, AIP Conference Proceedings, American Institute of Physics: 1975; pp 51-52.
11. Chen, J.-L.; Kaltsoyannis, N., DFT+ U study of uranium dioxide and plutonium dioxide with occupation matrix control. *The Journal of Physical Chemistry C* **2022**, *126*, 11426-11435.
12. Tran, F.; Blaha, P., Implementation of screened hybrid functionals based on the Yukawa potential within the LAPW basis set. *Physical Review B* **2011**, *83*, 235118.
13. Bylander, D.; Kleinman, L., Good semiconductor band gaps with a modified local-density approximation. *Physical Review B* **1990**, *41*, 7868.
14. Heyd, J.; Scuseria, G. E.; Ernzerhof, M., Hybrid functionals based on a screened Coulomb potential. *The Journal of chemical physics* **2003**, *118*, 8207-8215.
15. Krukau, A. V.; Vydrov, O. A.; Izmaylov, A. F.; Scuseria, G. E., Influence of the exchange screening parameter on the performance of screened hybrid functionals. *The Journal of chemical physics* **2006**, *125*, 224106.
